# Effects of high-pressure homogenization and ultrasound on the composition, structure, and physicochemical properties of proteins extracted from *Nannochloropsis Oceania*

**DOI:** 10.1016/j.ultsonch.2024.106851

**Published:** 2024-03-20

**Authors:** Wee Jian Cedric Sow, Juan Du

**Affiliations:** a Food, Chemical and Biotechnology Cluster, Singapore Institute of Technology, 10 Dover Drive, Singapore 138683, Singapore; b Department of Food Science, Purdue University, 745 Agriculture Mall Dr, West Lafayette, IN 47907, USA; c Sengkang General Hospital, Singapore Health Services, 110 Sengkang East Way, Singapore 544886, Singapore

**Keywords:** Microalgae, Alternative proteins, Emulsion, High-pressure homogenization, Ultrasound, Protein structure

## Abstract

This study examined the effects of high-pressure homogenization (HPH) and ultrasonication pre-treatment on the structural and physicochemical properties of proteins extracted from defatted *Nannochloropsis Oceania* biomass (DNOB). HPH treatment was found to enhance the solubility of protein extracted from DNOB compared to ultrasound, where samples pretreated with three passes (3P) of HPH exhibited lower solubility than two passes (2P). The morphology of extracted samples was visualized by scanning electron microscopy, which HPH pre-treatment, especially with more passes, were able to breakdown DNOB into fragments. Alternatively, more holes were displayed on the surface of the extracts pretreated with ultrasound especially when higher amplitude applied. The particle size of extracts from HPH_3P_ (129.5 µm) significant dropped from HPH_2P_ (314.25 µm), where samples pretreated with ultrasound at 20 % amplitude (US_20_) also decreased in particle size compared to 40 % amplitude (US_40_), from 115.25 µm to 78.22 µm. Protein flexibility of DNOB extracts were enhanced by both HPH_2P_ and HPH_3P_ but decreased for ultrasound samples. β-sheets were found to be the most abundant protein secondary structure for all samples, where samples treated with HPH_3P_ contained the highest percentage of β-sheets (72 %) than control, HPH_2P_, ultrasonication at 20 and 40 % amplitude (52–62 %). The high percentage of β-sheets found in HPH_3P_ sample also contributed to its outstanding emulsifying properties which stood out among all, especially at concentrations over 1 mg/ml. Results obtained from this study helped to direct the application of DNOB extracts as functional food ingredient for future food innovation.

## Highlights

• HPH 2 passes pre-treatment enhanced protein solubility but not for 3 passes.
• HPH caused the DNOB to be fragmented while ultrasound induced holes on the surface.
• Protein flexibility was enhanced by HPH treatment but decreased by ultrasound.
• Highest percentage of β-sheets was found in sample that underwent 3 passes of HPH.
• Emulsifying properties of samples underwent 3 passes of HPH stood out among all.

## Graphical abstract

**Figure:**
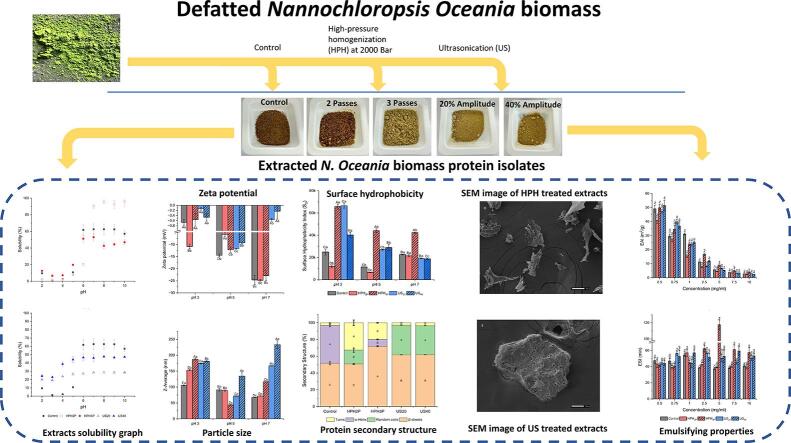

## 1 Introduction

Over the years the global population growth elevated food consumption and protein-rich foods have consistently been gaining popularity among consumers [1]. The United Nations Food and Agriculture Organization (FAO) predicted that meeting the food demands of over 9.3 billion people by 2050 will be a challenge [2] and the demand for food, particularly meat, is set to increase by 73 % to 455 million tons [3], [4]. Across different geographical regions, major sources of dietary proteins are derived from animal sources such as meat, eggs, and dairy products. Protein plays an essential role in the growth and maintenance of the human body, and as a macronutrient, they provide taste, texture, and flavors in food systems [5].

Microalga as unicellular, microscopic organisms have been considered an alternative, unconventional protein source and food supplement for animal and human nutrition, wastewater treatment, cosmeceutical and biofuels [6]. Though microalgae are a source of many beneficial compounds recommended for the prevention of metabolic problems related to aging, only few species are approved in the human diet list globally. A priority should be given to species of the genus *Nannochloropsis*, due to their suitability for intensive culture and high content of protein, high quality of poly-unsaturated fatty acid (PUFAs), antioxidants and some vitamins. The lipid, protein, and carbohydrates content of different *Nannochloropsis* isolates are found to be ranged from 28–45 %, 30–43 % and 15–35 % respectively [7]. *Nannochloropsis* is a unicellular alga of the class *Eustigmatophyceae*, found in both marine and freshwater. It is a small spherical (slightly ovoid) alga with cell membrane of 2–5 µm [8]. Plant-based surimi analogue incorporated with defatted *Nannochloropsis oceanica* was proved to enhance gel strength and *in vitro* protein digestibility [9].

The numerous membrane cell wall and barrier envelopes in *Nannochloropsis* post a challenge for extraction of biomolecules from microalgae such as protein isolation because these walls cannot be easily digested or degraded. *Nannochloropsis* cells comprise mainly carbohydrate in particular algaenan, a component that likely encompasses serval lipid-related species [10]. Algaenan is highly resistant to alkali/acid hydrolysis and aqueous/organic solubilization, and their biochemical characterization was under-explored due to the chemical alteration induced during the algaenan isolation process such as partly or entirely cleavage of (poly)esters as reported by Allard et al. [11]. Studies also shows that algaenan found in *Nannochloropsis* comprises long-chain aliphatic hydrocarbons that are subjected to ether cross-linking reactions [10]. Different extraction techniques and pre-treatment methods aimed at disintegration and degradation of membrane and complex cell walls were explored in the past, including physical (high-pressure homogenization, ultrasonication, manual grinding) and chemical treatments (acid and alkaline). High-pressure homogenization typically ruptures the cell or breaks it into several distinct parts and with more passes the cell debris may be degraded to smaller fragments [12]. Ultrasound is widely used in physical processing to extract protein because of its easy operation and mild conditions. It requires less energy, does not require the addition of beads, scalable to large volume, able to operate continuously and able to disrupt diverse range of agal species [13]. Study has shown that by combining both chemical and physical method as the physical method would consider as the pre-treatment of microalgae cell walls by breaking it and follow by chemical treatment as it showed increase in protein extraction [14]. Such treatment successfully improved the yield but not enough information on the techno-functional properties of protein extracts was reported. However, most of the previous studies focused on the improvement in yield, the effect of how pre-treatment extracted protein affects the protein functionalities and their application in food systems are under-explored.

Therefore, the objective of this study is to characterize the differences in composition, structural, morphological, and physicochemical properties of the protein extracted from defatted *Nannochloropsis Oceania* (DNOB) that were pre-treated under different physical treatment. High-pressure homogenization and ultrasound at different parameters were chosen as physical pre-treatment of DNOB, followed by alkaline-acid protein extraction process, in comparison to no pre-treatment involved protein extract as control.

## 2 Materials and methods

### 2.1 Materials

The DNOB were provided by Wintershine Pte. Ltd. (Singapore) and canola oil purchased from the local supermarket. Sodium hydroxide (NaOH) pellets, 37 % hydrochloric acid (HCl), sodium dodecyl sulfate (SDS), sodium chloride (NaCl), sodium acetate, sodium phosphate dibasic heptahydrate and sodium phosphate monobasic monohydrate were purchased from Sigma-Aldrich Pte. Ltd. (Singapore). 1-anilinonaphthalene-8-sulfonic acid (ANS) was purchased from Thermo Scientific, Invitrogen, USA.

### 2.2 Extraction of protein

#### 2.2.1 Physical pre-treatment

##### 2.2.1.1 High pressure homogenization

The pre-treatment of DNOB was performed with slight modification from Yassin et al. [15] where DNOB was dispersed (2 % w/v) in deionized water and well mixed, and the suspension was processed through a microfluidizer (M−110P, Microfluidics, United States) at 2000 bar for two and three passes, labelled as HPH_2P_ and HPH_3P_ respectively. Subsequently, mixtures were to undergo the same chemical treatment listed in section 2.2.2.

##### 2.2.1.2 Ultrasonication

The ultrasonication pre-treatment of DNOB was modified from Gerde et al. [16], where DNOB was dispersed (2 % w/v) in deionized water and mixed, and the suspension was surrounded in ice bath to prevent overheating. Samples were sonicated with ½ in. tip probe using a 20 kHz frequency ultrasound processor (Sonics Vibra Cell VCX 750, USA) for 20 min at 20 % and 40 % full amplitude (labelled as US_20_ and US_40_). Subsequently, mixtures were to undergo the same chemical treatment listed in section 2.2.2.

#### 2.2.2 Chemical extraction

The isolation of protein was performed with slight modification from Gerde et al. [16]. For the control sample (no pre-treatment), pre-weighted DNOB was suspended in deionized water to make a concentration of 2 % (w/v). The pH of all suspension solutions (control, HPH_2P_, HPH_3P,_ US_20_, and US_40_) were adjusted to pH 11.5 respectively using 2 M NaOH followed by stirring and heating at 60 °C for 5 h. Later, the samples were centrifuged (Sorvall X1R Pro, Thermo Fisher Scientific, Waltham, MA, USA) at 7,000 × *g*, 20 °C for 15 min while retaining the pellets (insoluble at pH 11.5). All the supernatants were combined and transferred into a beaker and chilled overnight in refrigerator at 4 °C. The pH of the supernatant was then adjusted to 3.2 with 2 M HCl and stirred for 30 min. The solution was then centrifuged at 20,000 × *g*, 4 °C for 15 min, with supernatant discarded, and pellets were retained and then suspended with deionized water. The pH of the obtained pellets was then adjusted to pH 7 using 2 M NaOH then freeze-dried (VirTis Benchtop Pro, SP Scientific, USA) at −80 °C under vacuum condition for 5 days for further analysis.

### 2.3 Proximate composition analysis

Total nitrogen content was determined using Gerhardt Dumatherm (C. Gerhardt GmbH & Co. KG, Königswinter, Germany) and the crude protein content was converted by N × 4.78 as a conversion factor [17], [18]. The total solid content was analyzed according to Osen et al. [19] with slight modification. Samples were dried to weight constancy at 105 °C in a thermogravimetric system (TG 209 F3 Tarsus®, NETZSCH, Germany) while the total ash content were determined in same system but at 900 °C with nitrogen protective airflow rate, 20 mL/min; and heating rate, 10 °C/min. The total lipid content was under detectable limit and therefore was assumed to be zero since the *Nannochloropsis Oceania* material was defatted when provided by Wintershine Pte. Ltd. Carbohydrate content was determined as the difference between 100 and the sum of the percentage of moisture, crude protein, and total ash contents with lipid is zero [9], [20].

### 2.4 Solubility

The determination of solubility of the extracted DNOB isolates was based on Zhao et al. [21] where 1 % (w/v) algae solution was prepared by dissolving the extracted protein isolates (Control, HPH_2P,_ HPH_3P_, US_20_ and US_40_) in 10 mM sodium acetate buffer, followed by being kept at 4 °C overnight to allow full hydration. Then 2 M HCl or 2 M NaOH was used to adjust the protein suspension to pH 2 – 12 and protein samples at each pH were centrifuged at 8000 × *g* for 15 min at 4 °C. The solubilized protein content in collected supernatants was determined by Pierce™ Modified Lowry Protein Assay Kit (Thermo Scientific™, Rockford, USA) using bovine serum albumin as calibration standard. Additionally, the total protein content was determined using Kjeldahl method mentioned in section 2.3. The solubility of the algae was calculated as followed in equation (1).

(1) $$Solubility\left[\%\right]=\frac{Protein\;concentration\;of\;supernatant}{Total\;protein\;concentration.}\times 100\%$$

### 2.5 Zeta potential

Zeta potential was determined according to Zhao et al. [21] where 0.1 % (w/v) algae solution was prepared by dissolving the extracted protein powders (Control, HPH_2P_, HPH_3P_, US_20_ and US_40_) in 10 mM sodium acetate buffer. The pH of the protein solutions was then adjusted to 7 with 2 M HCl or 2 M NaOH prior to measurement. The solution was then filtered with a 0.45 µm cellulose-acetate filter and zeta-potential of the protein solutions was measured using a nanoparticle analyzer (Nanosizer SZ-100, Horiba Ltd., Kyoto, Japan).

### 2.6 Particle size distribution

The particle size expressed as hydrodynamic diameter (z-average) of all the extracted DNOB in D.I water at 0.1 % (w/v) were determined with dynamic light scattering (DLS) according to Du et al. [22] using a nanosizer (Nanosizer SZ-100, Horiba Ltd., Kyoto, Japan) with scattering angle of 90°. D_4,3_ and D_3,2_ of the samples listed in Table S2 were determined according to Yassin et al. [15] with slight modification. Refractive indexes for the sample and dispersant were 1.37 for *Nannochloropsis* and 1.33 for water respectively [23]. Each measurement was conducted in triplicate.

### 2.7 Surface hydrophobicity

The surface hydrophobicity was determined according to a method of Kato and Nakai [24] with slight modification. Stock solution of 8 × 10^-3^ M ANS were prepared in 0.1 M phosphate buffer (pH 7.4) and were stored at room temperature in a centrifuge tube wrapped in aluminum foil to avoid exposure to light. 20 µL of the ANS solution was added to 4 mL of diluted protein sample with concentration of 0.125, 0.25, 0.5, 0.75 and 1 mg/mL and set the samples 10 min in the dark. Subsequently, the fluorescence intensity was measured with excitation and emission wavelength of 390 and 470 nm respectively with microplate reader (Synergy™ HTX Multi-Mode, BioTek USA). The surface hydrophobicity index, S_0_ was calculated from the regression slope, or net relative fluorescence intensity vs protein concentration [25]. The analysis was conducted in triplicate.

### 2.8 Fourier transform infrared spectroscopy (FTIR)

All lyophilised protein samples obtained from Section 2.2 were sent to an attenuated total reflectance-Fourier transform infrared (ATR-FTIR) spectrometer (Agilent Cary 630 FTIR, Agilent Technologies, USA) for protein secondary structure characterisation. Spectrum of each sample within the range of 600–4000 cm^−1^ was recorded after 64 scans with a resolution of 4 cm^−1^, in which the amide I region (1700–1600 cm^−1^) underwent further deconvolution by OriginPro software (OriginPro, Version 2023b. OriginLab Corporation, Northampton, MA, USA.), under the function of Gaussian curve fitting [26]. The proportion of each secondary structure α-helix (1650–1660 cm^−1^), β-sheet (1610–1640 & 1670–1680 cm^−1^), β-turn (1660–1670 & 1680–1700 cm^−1^), and random coil (1640–1650 cm^−1^) was calculated from the relative integrated areas of each component peak.

### 2.9 Protein flexibility

The protein flexibility was measured according to the method described by Kato et al. [27] and Yan et al. [28] with slight modifications. The extracted protein samples were dissolved in 0.05 mol/L tris-HCL (pH 7.5) to make protein solutions with a concentration of 0.5 mg/mL, then trypsin (potency ≥ 2500 units/mg) was added at 1:16 ratio by volume to the sample solution). The mixture was then well mixed and incubated at 38 °C for 10 min followed by centrifugation at 5000x*g* for 30 min, the absorbance of the supernatant was measured with Modified Lowry Protein Assay Kit by Pierce™ (Thermo Scientific™, Rockford, USA) at wavelength of 750 nm. The absorbance value was used to quantitatively express protein flexibility.

### 2.10 Scanning electron microscope

5 mg of the extracted samples were scattered onto a conductive adhesive tape and subsequently coated with platinum for 60 s at a flowrate of 20 mA. The sample was then viewed in the vacuumed stage in the microscopic chamber of a scanning electron microscope (JSM IT800, Jeol Asia, Tokyo, Japan) [23].

### 2.11 Emulsifying activity index and emulsion stability index

The Emulsifying Activity Index (EAI) determination method were based on Tan et al. [29] with slight modifications. Briefly, 300 µL of canola oil was added to 3 mL of protein solution concentration range from 0.1 % to 1 % and homogenized using the IKA Homogenizer (IKA ULTRA-TURRAX T18 digital, Germany) with S18N-10G dispersion tool at 21,000 rpm for 1 min. A total of 30 µL of the emulsion was then added to 3 mL of 0.1 M NaCl in 0.1 % (w/v) SDS solution and mixed well using a vortex mixer (Vortex-Genie 2: Scientific Industries, New York, USA). The absorbance was read at 500 nm, using a UV–vis spectrophotometer (UV5 Bio, Mettler Toledo, Switzerland). The EAI (Equation (2) were calculated as follows:

(2) $$EAI\left(\frac{m^{2}}{g}\right)=\frac{2T}{c(1-\Phi )}$$

where T = turbidity (2.303) × (dilution factor (1 0 0) × absorbance ÷ pathlength (0.01 m), c = protein concentration (g/m^3^) and Φ = oil volume fraction (0.1). The calculation of Emulsion Stability Index (ESI) (Equation (3)) was based on [30] using the following formula:

(3) $$ESI\left(min\right)=\frac{A_{0}}{\left(A_{0}-A_{1}\right)}\times \Delta T$$

where A_0_ = absorbance at zero minutes, and A_1_ = absorbance at 30 min.

### 2.12 Statistical analysis

Statistical analyses were performed with one-way analysis of variance (ANOVA) test using Minitab software (Minitab® 21.4.1). The means and standard deviation were compared using Tukey’s honest significance test at 95 % confidence level, where a significant difference is observed when p < 0.05. Analytical values of experiments are shown as mean ± standard deviation.

## 3 Results and discussion

### 3.1 Proximate composition analysis

Table S1 shows the proximate composition of protein powders extracted from DNOB with different pre-treatment methods (control, HPH_2P_, HPH_3P_, US_20_ and US_40_). It was depicted that HPH treated DNOB extracts had a lower protein content (55.00 % and 54.79 % for HPH_2P_ and HPH_3P_), as compared to control (58.39 %). This suggested that using HPH might not necessarily lead to a higher purity of protein but instead with these high shear and pressure mechanisms could cause protein aggregation and changes in the cellulose structure of DNOB which resulted the increases of carbohydrate content from 24.03 % to 25.52 % and 31.19 % for control, HPH_2P_ and HPH_3P_, respectively [31]. Likewise, lower ash content was observed with increased number of passes for HPH from 11.90 % to 7.47 % but with the drop in the ash content did not contribute to higher protein content but carbohydrate content instead which indicated that both ash and carbohydrate were inversely proportional to each other.

On the other hand, ultrasound treated protein extracts (US_20_ and US_40_) protein content had slight increased (57.17 % and 59.15 % for US_20_ and US_40_) but only at higher amplitude of 40 %, while the moisture and ash contents were statistically similar to control. Also, it was observed that the protein content and amplitude applied to the treatment are directly proportional to each other while inversely proportional for carbohydrate content [32].

### 3.2 Solubility

**Fig. 1:**
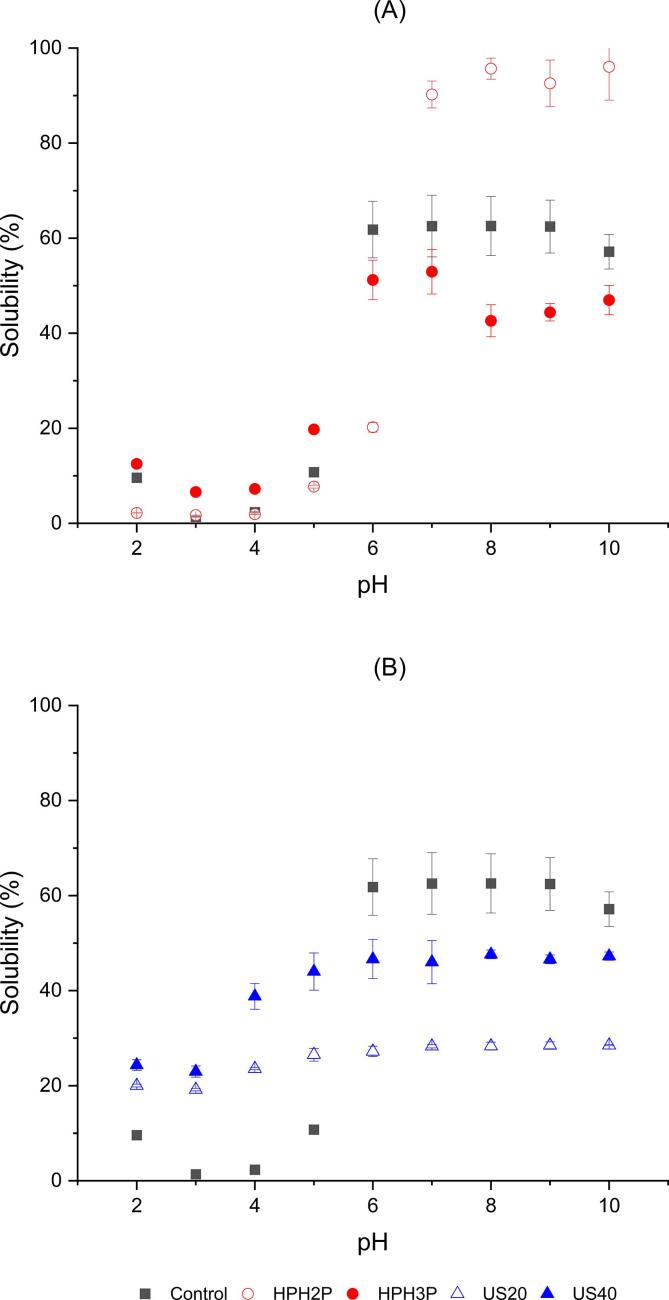
Solubility as a function of pH of **(A)** DNOB extracts for control, samples pre-treated with high-pressure homogenization of 2 passes (HPH_2P_) and high-pressure homogenization of 3 passes (HPH_3P_). and **(B)** control, samples pre-treated with ultrasound at 20% amplitude (US_20_) and ultrasound at 40% amplitude (US_40_).

The solubility of control, HPH_2P_, HPH_3P_, US_20_ and US_40_ were shown in Fig. 1. All the samples had minimum solubility near a pH of 3, which corresponded to their isoelectric point [16]. The minimum solubility which occurs near the isoelectric point was due to the absence of net charge on the proteins, thus no repulsive interactions and the protein-proteins interaction, which reduces the solubility [33]. Higher solubility above pH 6 were observed, as the pH was away from the isoelectric point, when there were more negative charges on the surface of the proteins which caused repulsive forces that tend to overcome the hydrophobic interaction of proteins [34].

As observed in Fig. 1A, at higher pH (6 and above), solubility of HPH_2P_ was the highest followed by control then HPH_3P_. This indicated that protein extracts treated with 3 passes of HPH did vary from control which had no physical treatment and samples underwent 2 passes. This was due to the cell disruption effect caused by HPH process which involved sudden formation of turbulence, shear stress and cavitation, and the mechanical induced movement of the suspension being forced to go through a small-sized orifice [35]. This treatment caused the breakage of protein aggregates and increased its solubility, or unfolding of proteins, which increased the interaction between protein and water thus, increasing the solubility [36].

In contrast, both US_20_ and US_40_ exhibited lower solubility as compared to control. It was observed that with higher amplitude treated samples had higher solubility based on the results indicated in Fig. 1B. This was due to the cavitation from shear forces, shock waves and impingement resulting from water jet at a solid and liquid interface that may alter the structures of protein leading to increased solubility [37]. However, in this study, it was demonstrated that higher amplitude had lesser effect in the solubility which could be due to the secondary structure presented in Fig. 4A that no α-helix was to be found for ultrasonicated samples and Tan et al. [38] also reported that α-helix structures were identified as the main correlation factors for solubility.

### 3.3 Scanning electron microscopy

**Fig. 2:**
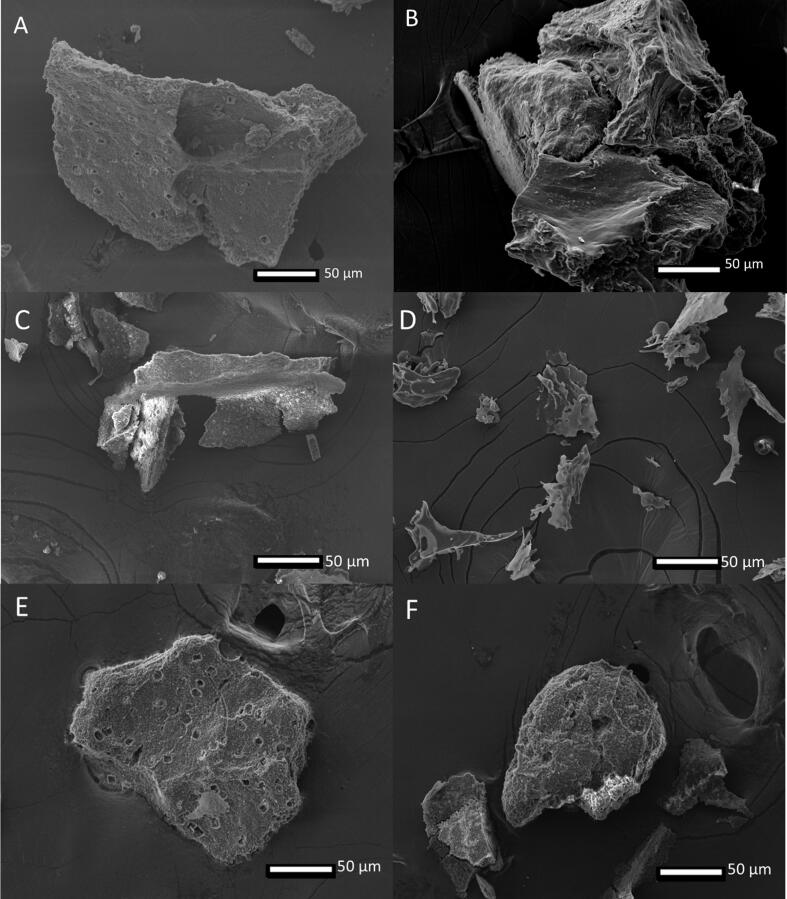
Morphological images of native **(A)**, control **(B)**, high-pressure homogenization of 2 passes (HPH_2P_) **(C)** and high-pressure homogenization of 3 passes (HPH_3P_) **(D)**, ultrasound at 20 % amplitude (US_20_) **(E)** and ultrasound at 40 % amplitude (US_40_) **(F)** detected by scanning electron microscopy (SEM) at 350 × magnification.

As shown in Fig. 2A, the native DNOB appeared to have smooth, rocky-like structures while Fig. 2B, untreated DNOB extracts contained cell debris which had irregular, uneven, rough surfaces, and tiny holes, unlike the treated samples which depicted a certain degree of disruption on the surface of the cell debris from the alkaline-acid protein extraction process. While the HPH pre-treated DNOB extracts containing debris showed cracks on the surface, changes in overall structure from a circular-like shape to elongate-like fragmented (Fig. 2C & D) and size reduction was observed which agreed with results found in Table S2. The volume-weighted mean particle size (D_4,3_) of HPH_2P_ (314.25 µm) was indifferent from control (283.3 µm) but dropped in HPH_3P_ (129.5 µm). The area-weighted mean D_3,2_ for HPH_2P_, HPH_3P_ and control were insignificantly different. This indicates that HPH treatment was only able to cause significant DNOB breaking down into smaller debris when 3 or more passes applied [39].

On the other hand, extracts treated with ultrasonication were found to have cell debris containing holes on the surfaces depicted in Fig. 2E & F and size reduction in D_4,3_ (115.25 µm and 78.22 µm for US_20_ and US_40_) and D_3,2_ (0.81 µm and 0.68 µm for US_20_ and US_40_) were observed compared to control (D_4,3_ of 238.25 µm and D_3,2_ of 7.13 µm), as indicated in Table S2. The holes present on the surface of the cell debris were resulted from the cavitation shear forces and shock waves as reported by Lee et al. [37]. The differences in amplitude applied did have an impact on the structure surfaces as lower amplitude tend to generate larger and lesser holes, while higher amplitude resulted in smaller and more holes on the surface which was also reported by Mohagheghian et al. [40].

### 3.4 Zeta potential, dynamic light scattering and surface hydrophobicity

**Fig. 3:**
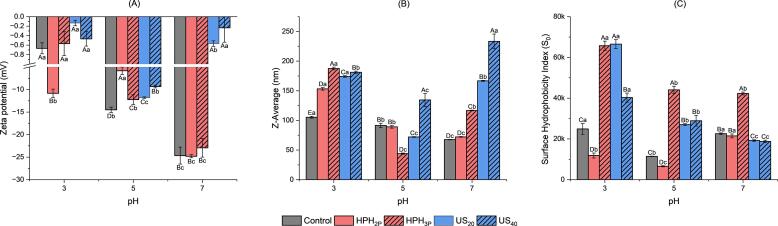
Zeta potential **(A)**, hydrodynamic size (Z-average) **(B)** and surface hydrophobicity index (**C**) of DNOB extracts for control, high-pressure homogenization of 2 passes (HPH_2P_), high-pressure homogenization of 3 passes (HPH_3P_), ultrasound at 20 % amplitude (US_20_) and ultrasound at 40 % amplitude (US_40_) at different pH conditions of 3, 5 and 7. Uppercase letters indicate significant differences within the same pH while lowercase letters indicate significant differences within the same treatment (p < 0.05).

Fig. 3 presented the zeta potential, particle size and the surface hydrophobicity of control, HPH_2P_, HPH_3P_, US_20_ and US_40_. Observation from Fig. 3A showed that at pH 3, all samples exhibited close to zero charge since the pH is near isoelectric point with the exception of HPH_2P_ which had negative charges. Interestingly, at a neutral pH of 7, the zeta potential of ultrasound treated samples US_20_ and US_40_ had gone back close to zero, which exhibited similar situation when the samples were at its isoelectric point, and this indicated that the surface charges of extracts with ultrasound pre-treatment were neutrally charged at pH of 7, but the rest of the samples were more negatively charged. As shown in Fig. 3B, all the samples were statistically significantly different at pH of 3, while at both pH 5 and 7 only control and HPH_2P_ were not significantly different while US_40_ were the highest among all. Another trend was observed that when the pH increased, the z-average values of both control and HPH_2P_ decreased. In contrast, the z-average value of HPH_3P_, US_20_ and US_40_ decreased and increased again. This was due to the electrostatic interaction between the protein particles and particle size of the protein which was affected by its conformation ensemble and electrostatic effect reported by Chin et al. [41]. As observed in Fig. 3A, the zeta potential values for US_20_ and US_40_ at pH of 3 and 7 were close to zero and in Fig. 3B, the z-average values were significantly higher than the rest of the samples, indicating for ultrasound treated samples, the zeta potential was directly proportional to z-average values, as the extracted proteins aggregated with surface charge neutralized.

Fig. 3C described the surface hydrophobicity index (S_0_) of samples as a function of pH. At pH of 3, S_0_ of control, HPH_2P_ and HPH_3P_ were significantly different from each other, and it was observed that there was an effect on the surface hydrophobicity index with different parameters of HPH treatment. There was a drop in surface hydrophobicity when the sample was treated with HPH of 2 passes and followed by a drastic increase in surface hydrophobicity once treated with additional passes from 2 to 3. For ultrasound treated samples (US_20_ and US_40_), the surface hydrophobicity increased, and differences between US_20_ and US_40_ were only observed at pH of 3. Furthermore, similar trends were observed for S_0_ values at pHs of 5 and 7. Lower surface hydrophobicity were observed with increased pHs for most of the samples, with the lowest S_0_ values for US_20_ and US_40_ pH of 7. Zhu et al. [42] reported that low α-helix and high β-sheet contents in the protein structure suggested simultaneous increase in the surface hydrophobicity index as observed in Fig. 5A. Surface hydrophobicity is an important index to characterize the emulsifying properties of proteins and is related to their conformations and flexibilities [43], which were further discussed in sections of 3.5 to 3.7.

### 3.5 FTIR analysis and secondary structure

**Fig. 4:**
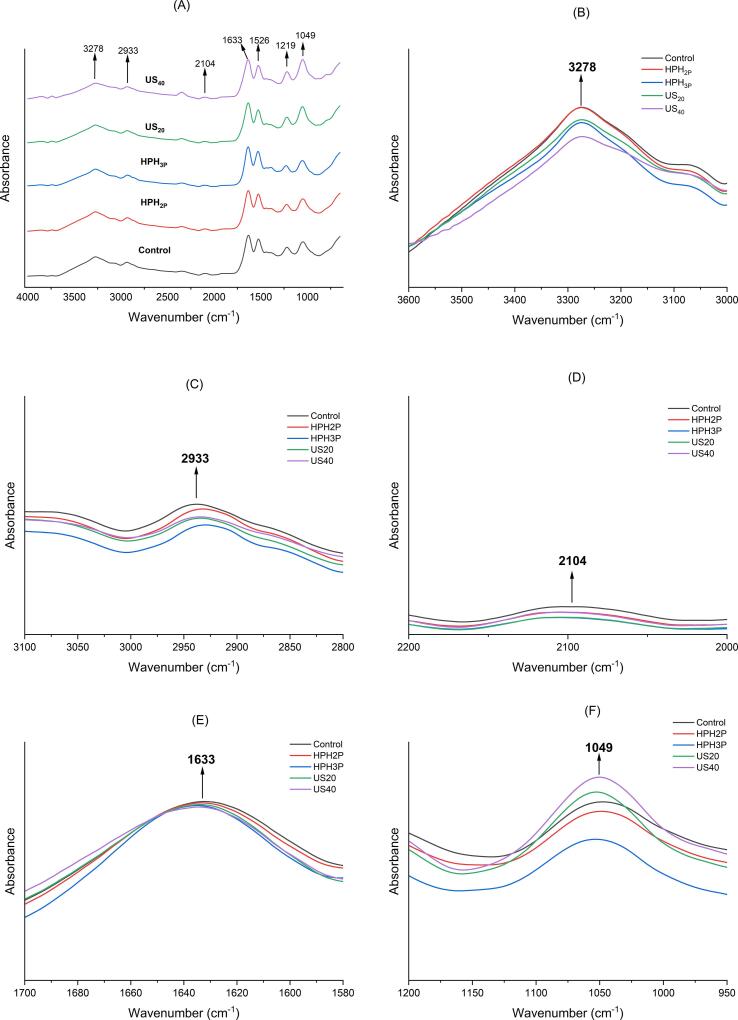
Stacked FTIR spectra (500 – 4000 cm^−1^) of DNOB extracts with physical pre-treatment of high-pressure homogenization and ultrasound pre-treatments and without physical pre-treatment **(A)**, OH group in cellulose at 3200 – 3500 cm^−1^**(B)**, algaenan peak at 2800 – 3020 cm^−1^**(C)**, Alkyne peak at 2100 – 2260 cm^−1^**(D)**, vibration of water molecules absorb in cellulose **(E)**, C – O – C bond peak present at 950 – 1200 cm^−1^**(F)**.

Fig. 4A-F depicted the FTIR spectra of the DNOB extracts with different pre-treatment, where absorbance peaks appeared in 3200–3500 cm^−1^ (OH group in cellulose) where Scholz et al. [44] and Brown [45] reported that cellulose was the majority of carbohydrate found in the Nannochloropsis. Algaenan, encompasses disparate types of enzymatically and chemically resistant aliphatic material, was also found at the region of 2800 – 3020 cm^−1^ with prominent peak at 2933 cm^−1^ which was consistent of what was found in a previous study done by Scholz et al. [44]. They also reported that Nannochloropsis comprise straight-chain (∼C_30_), which was highly saturated aliphatic compound joined by ether bond at terminal and one or two midchain position which indicated a peak at 2101 cm^−1^ (Alkyne – triple bond carbon). Another noticeable peak at 1633 cm^−1^ of which indicated vibration of water molecules absorbed in cellulose reported by Binte Abdul Halim et al. [23] and confirmation of glucose appearance reported by De Paula et al. [46] and Pu et al. [47]. Strong absorption signals were observed at 1049 cm^−1^, which were attributed to the C-O-C band, indicating presence of polysaccharides in the range of 950–1200 cm^−1^ [48].

**Fig. 5:**
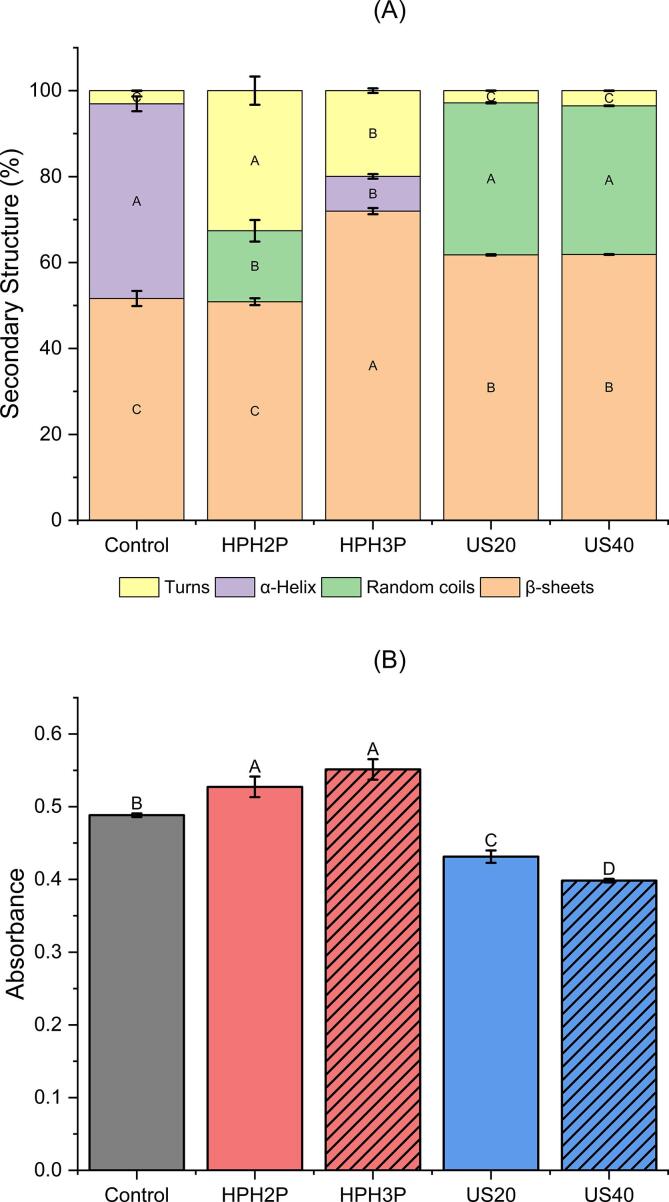
The percentage of protein secondary structure **(A)** in DNOB extracts for control, physical pre-treatment with high-pressure homogenization of 2 passes (HPH_2P_), high-pressure homogenization of 3 passes (HPH_3P_), ultrasound at 20 % amplitude (US_20_) and ultrasound at 40 % amplitude (US_40_). Average values within the same protein secondary structure by different samples represented by different letters are significantly different (p < 0.05). DNOB extracts flexibility expressed in absorbance **(B)** in DNOB extracts for control, physical pre-treatment with high-pressure homogenization of 2 passes (HPH_2P_), high-pressure homogenization of 3 passes (HPH_3P_), ultrasound at 20 % amplitude (US_20_) and ultrasound at 40 % amplitude (US_40_). Values with different letters are significantly different at (p < 0.05).

However, absorption peak at 1633 cm^−1^ was seen for all samples and was essentially assigned to a C = O stretching (amide I) and corresponded to the protein β-sheets structure (1623–1641 cm^−1^) which was consistent with the secondary structure composition in Fig. 5A. The appearance of band at 1219 cm^−1^ for all samples was a result of the absorption of N-H bending vibration (amide III) and corresponded to β-sheets (1181–1248 cm^−1^) [34].

As depicted in Fig. 5A, the β-sheet was the most abundant secondary structure of all the samples, notably HPH_3P_ that had the highest content. For samples underwent HPH pre-treatment, an increase in β-sheet was observed in HPH_3P_ but not in HPH_2P_ as compared to control. Additionally, α-helix structure was absent in HPH_2P,_ but random coils was observed instead. With increasing number of passes to 3, existence of α-helix was found but with lower amount as compared to control while the random coil was not present. There were increment of turns for both HPH_2P_ and HPH_3P_ but as the number of passes increased, the turns tend to decrease as observed.

The β-sheet contents increased in the ultrasound treated samples of US_20_ and US_40_ compared to control, which was agreeable with the study by Yan et al. [28], that ultrasound was able to alter the protein secondary structure in content of α-helix and random coils but not turns. The decrease in α-helix content was related to the partial unfolding of the α-helical region caused by ultrasound cavitation [49]. The increase in the random coils was attributed to the transformation of α-helix into random coils, as ultrasound treatment reduced the amount of intramolecular hydrogen bonding [50] causing the unfolding of protein molecules by cavitation generated by the ultrasound which later formed random coil structure [28], [51].

### 3.6 Protein flexibility

In Fig. 5B, HPH treated samples did have a significant increase in protein flexibility compared to control, and the increase of protein flexibility correlated to the increasing number of passes of HPH. When subjected to 3 passes, HPH_3P_ exhibited the highest flexibility which was consistent with the changes in the secondary structure, including the increase of β-sheet and decrease of turns in Fig. 5A.

In contrast, ultrasound treated samples decreased on their protein flexibility compared to both HPH and control. As mentioned in [28] protein flexibility was not positively correlated to the increase in the ultrasound power and in this study, it was observed that the amplitude of ultrasound (20 % and 40 %) and the protein flexibility had an inversely proportional relationship. The shear force and cavitation introduced by HPH treatment disrupted the hydrogen bonding or hydrophobicity of the protein, leading to the destruction of the rigid protein structure and increased in its flexibility [28]. In contrast, both [37] and [28] reported that high ultrasound power led to excess exposure of hydrophobic groups which later re-associated or aggregated to form more stable structures and decrease its protein flexibility. Lastly, protein flexibility was related to surface hydrophobicity, solubility, and the main chain structure of the polypeptide chain and thus, it was necessary to further analyse the corelation between protein flexibility, structure, and functionality [52].

### 3.7 Emulsifying properties

**Fig. 6:**
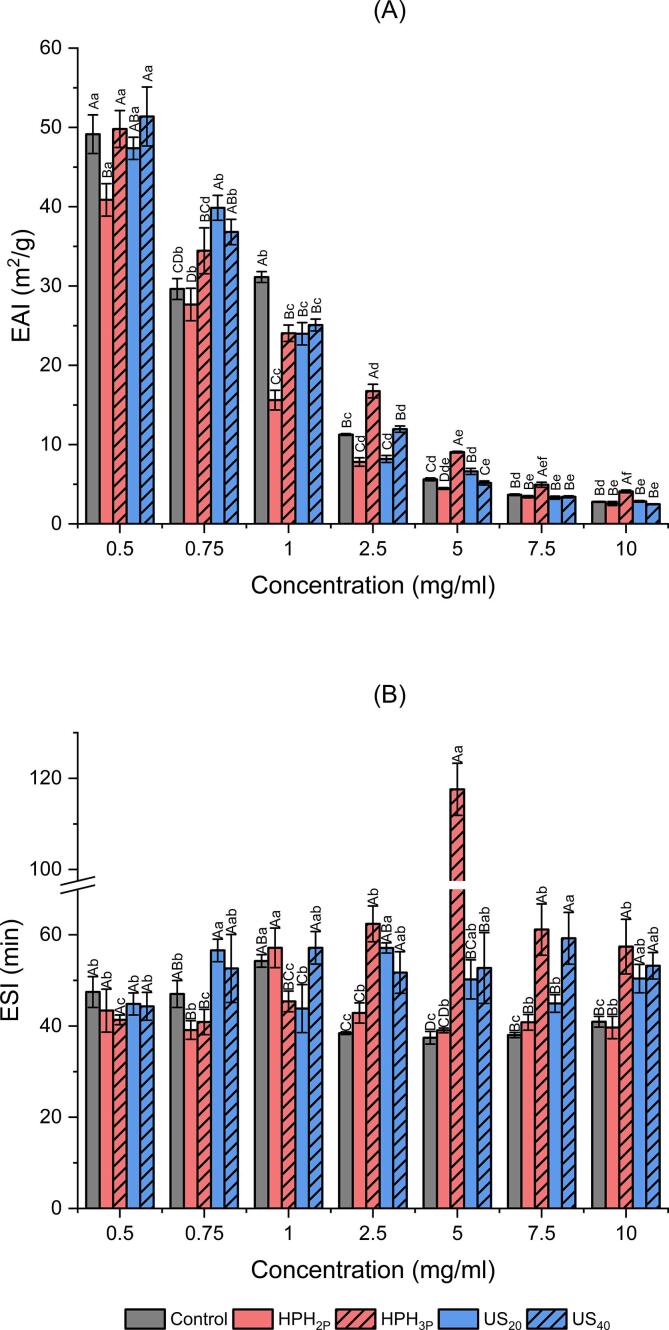
Emulsifying activity index values **(A)** and emulsifying stability index values **(B)** of emulsion prepared with DNOB extracts at concentration ranges from 0.5 to 10 mg/ml for control, samples pre-treated with high-pressure homogenization of 2 passes (HPH_2P_), high-pressure homogenization of 3 passes (HPH_3P_), ultrasound at 20 % amplitude (US_20_) and ultrasound at 40 % amplitude (US_40_). Uppercase letters indicate significant differences within the same concentration while lowercase letters indicate significant differences between the same treatment (p < 0.05).

As shown in Fig. 6A, HPH_2P_ had the lowest EAI values among all samples at concentrations less than 1 mg/ml while control, HPH_3P_, US_20_ and US_40_ performed better. Noticeable that EAI values were higher at lower concentrations due to the formulation of EAI (Equation (2) as EAI value was inversely proportional to protein concentration. However, the trend of EAI values started to change when the concentration increased (2.5 mg/ml and above), it was observed that EAI values of HPH_3P_ were significantly higher than the rest. The findings in Fig. 5A and B indicated that the percentage of β-sheet in secondary structure and protein flexibility of HPH_3P_ were the highest among all which agreed with the finding of others. [53], [54], [55] had reported that changes in secondary structure such as increase in β-sheets and decrease in α-helix improved emulsifying properties and increased in protein flexibility, which resulted in increasing interaction between protein and lipids as well as protein rearrangements at the oil–water interface.

Likewise, the ESI results shown in Fig. 6B exhibited similar trend as EAI, that HPH_3P_ had the highest ESI value of concentration above 1 mg/ml. Fig. 3C and Fig. 5B had indicated that the values of protein surface hydrophobicity and flexibility of HPH_3P_ were the highest among all. Previous studies had shown that increase in protein flexibility [42] and surface hydrophobicity [56] were able to enhance the ability of protein to stabilize the oil–water interface. At concentration above 1 mg/ml, ultrasound treated samples of US_20_ and US_40_ performed better than control and HPH_2P_ but HPH_3P_ had the highest ESI value among all. This finding agreed with what was reported in a study by Yan et al. [28] that ultrasound treatment did improve the emulsifying properties of soybean protein isolate. In general, the EAI and ESI results demonstrated that HPH and ultrasound as physically induced pre-treatments were able to influence the emulsifying properties of the protein extracts. Moreover, the physical processing parameters of these pre-treatments were also important in altering the protein structure and emulsifying properties, as three passes of HPH were able to elevate the content of β-sheet in the secondary structure of protein, resulting in better protein flexibility and surface hydrophobicity which subsequently enhanced the protein emulsifying properties. When it comes to ultrasound pre-treatment, the EAI and ESI value of samples (US_20_ and US_40_) were similar for most of the concentrations. It was reported that excessive power of ultrasound did not help in the emulsifying properties as the protein may aggregate again through hydrophobic interactions and therefore, a balance between exposure of hydrophobic group and aggregation of protein was needed [57].

## 4 Conclusion

HPH and ultrasound as physical pretreatment had played a critical role in altering the protein extracts structural conformations which attributed to the dissimilarity of the physiochemical and emulsifying properties. It was observed that three passes of HPH had a decreasing effect in protein solubility as compared to two passes and control. For ultrasound pretreatment, it did not enhance the solubility of extracted protein isolates compared to control, but it was deduced that with increasing amplitude of the ultrasound, the solubility was slightly elevated. Also, HPH with increased number of passes did enhance the surface hydrophobicity index value, the percentage of β-sheet in the protein secondary structure, and protein flexibility while on the other hand, ultrasound pretreatment samples resulted in removal of α-helix structure with no differences in surface hydrophobicity index but a slight decrease in protein flexibility between US_20_ and US_40_. HPH also modified the morphology and particle sizes of the DNOB extracts, with three passes of HPH, the DNOB extracts contained more fragmented structure and smaller particles as compared to two passes of HPH with larger particle. While higher amplitude ultrasound treatment was able to induce more numbers and reduce particle size but smaller sized holes on the surface of the protein extracts. Another observation noted was that three passes of HPH pretreatment enhanced the emulsifying properties of protein extracts especially at concentrations of more than 1 mg/mL while no significant differences were observed for US_20_ and US_40_. Although results from this study gave us some insight and help to navigate the use of DNOB as an alternative protein towards human food system, further evaluation is needed to understand the composition such as carbohydrate as the isolates still consist about 24–30 % of carbohydrate. Future studies can focus on the direction of how the carbohydrate plays a part in the protein functionalities as these physical pretreatments might release various form of carbohydrate in the process of isolation.

## CRediT authorship contribution statement

**Wee Jian Cedric Sow:** Writing – review & editing, Writing – original draft, Visualization, Software, Methodology, Investigation, Conceptualization. **Juan Du:** Writing – review & editing, Supervision, Project administration, Methodology, Funding acquisition, Conceptualization.

## Declaration of competing interest

The authors declare that they have no known competing financial interests or personal relationships that could have appeared to influence the work reported in this paper.
