# Supplementary material for: Effects of high-pressure homogenization and ultrasound on the composition, structure, and physicochemical properties of proteins extracted from Nannochloropsis Oceania
Source: Ultrason Sonochem. 2024 Mar 20;105:106851. doi: 10.1016/j.ultsonch.2024.106851 (PMC10981087; doi:10.1016/j.ultsonch.2024.106851)
Supplement: Supplementary data 1 [file mmc1.docx]

**Table S1.**

The proximate composition and physical appearance of DNOB extracts used in our study.

| Constituents | Control | HPH_2P_ | HPH_3P_ | US_20_ | US_40_ |
| --- | --- | --- | --- | --- | --- |
| Protein (N x 4.78) (%) | 58.39 ± 2.76^ab^ | 55.00 ± 0.70^b^ | 54.79 ± 0.32^b^ | 57.17 ± 0.88^ab^ | 59.15 ± 0.75^a^ |
| Moisture (%) | 8.03 ± 0.62^a^ | 7.56 ± 0.61^a^ | 6.53 ± 0.82^a^ | 6.81 ± 0.42^a^ | 7.59 ± 0.55^a^ |
| Ash (%) | 9.54 ± 2.02^ab^ | 11.90 ± 0.34^a^ | 7.47 ± 0.28^b^ | 10.7 ± 1.87^ab^ | 10.95 ± 0.96^ab^ |
| Carbohydrate (%) | 24.03 ± 4.06^b^ | 25.52 ± 0.98^ab^ | 31.19 ± 1.03^a^ | 25.31 ± 1.77^b^ | 22.30 ± 1.41^b^ |
|  | 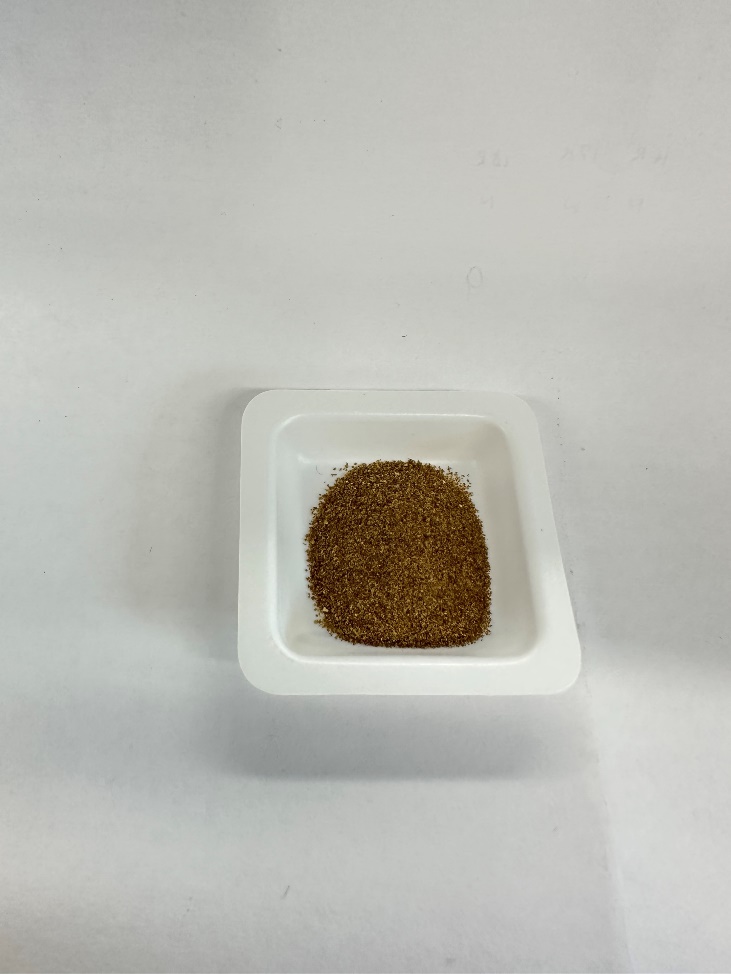 | 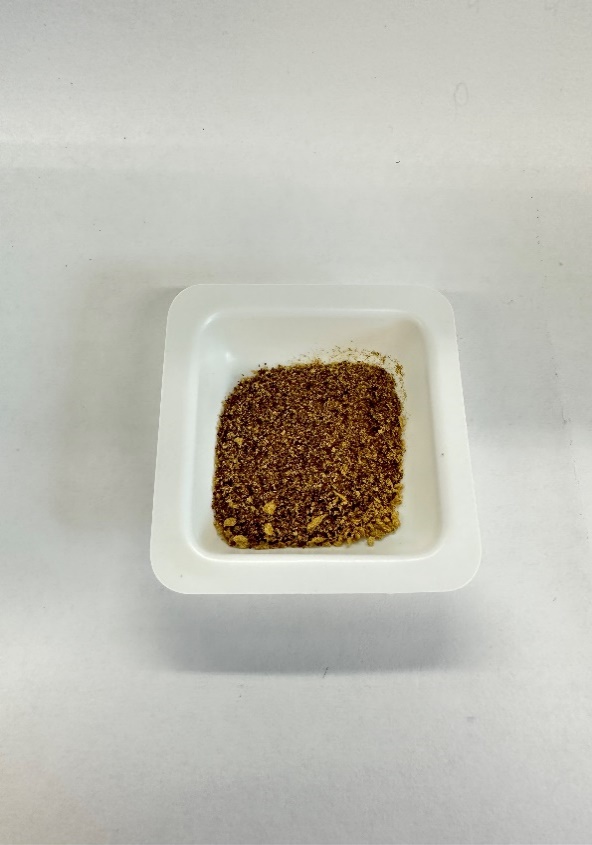 | 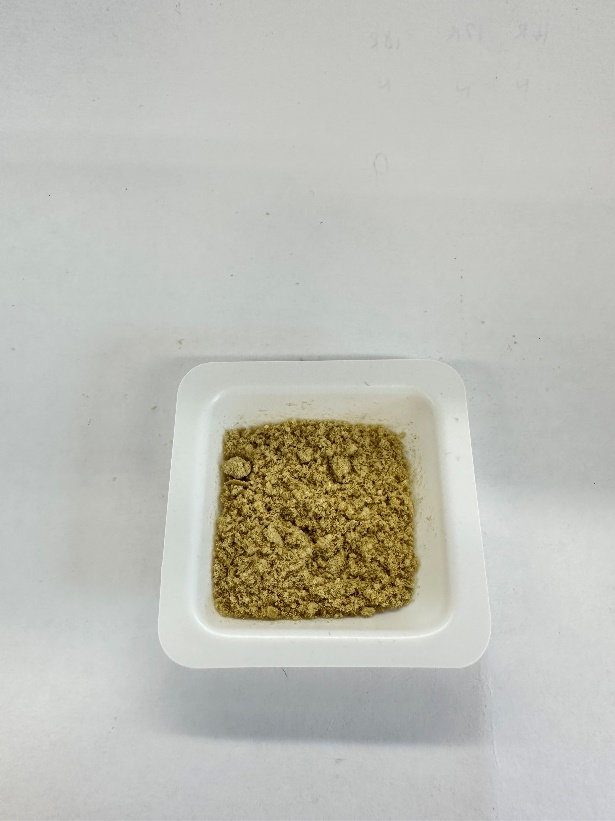 | 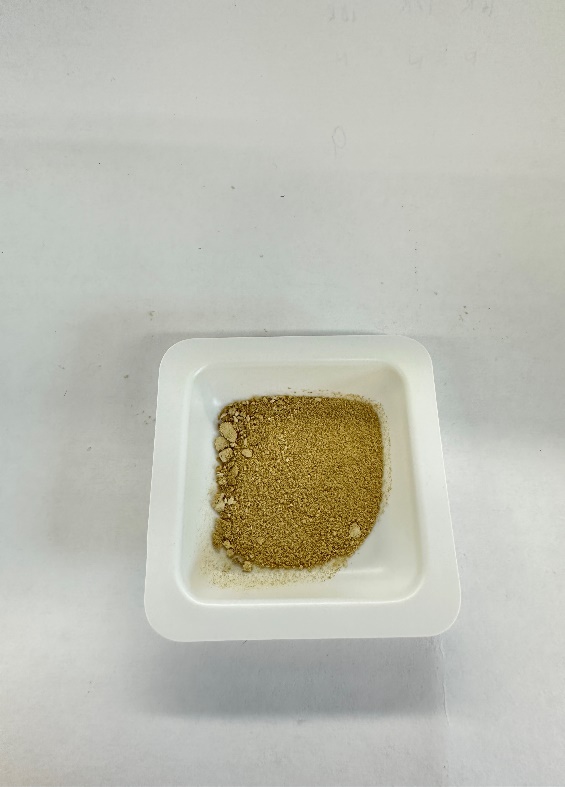 | 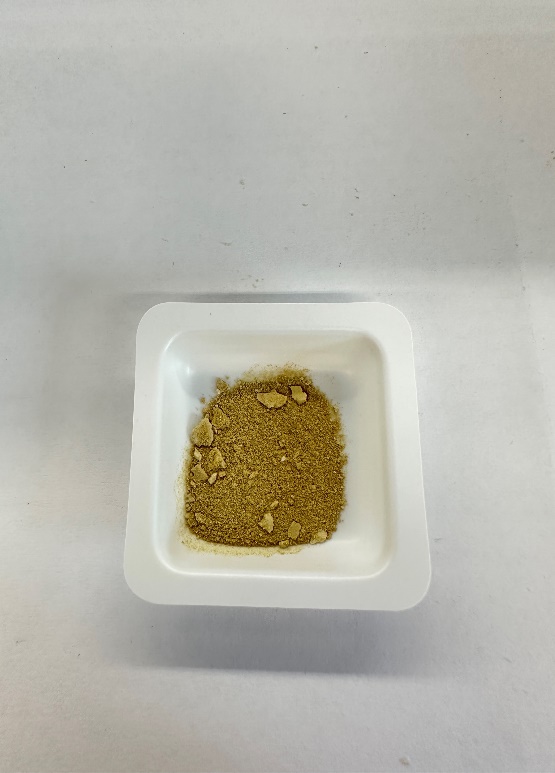 |

**Table S2.** Particle size in D_4,3_ and D_3,2_ of extracts in D.I water with different treatments.

|  | Native | Control | HPH_2P_ | HPH_3P_ | US_20_ | US_40_ |
| --- | --- | --- | --- | --- | --- | --- |
| D_4,3_ (µm) | 653.0 ± 6.6**^a^** | 283.3 ± 2.2**^b^** | 314. 5 ± 2.9**^b^** | 129.5 ± 6.8**^c^** | 115.35 ± 2.9**^c^** | 78.2 ± 4.8**^d^** |
| D_3,2_ (µm) | 347.0 ± 5.5**^a^** | 7.13 ± 0.08**^bc^** | 11.25 ± 0.06**^b^** | 12.27 ± 1.15**^b^** | 0.81 ± 0.01**^c^** | 0.68 ± 0.01**^c^** |
